# Predictors of indoor absolute humidity and estimated effects on influenza virus survival in grade schools

**DOI:** 10.1186/1471-2334-13-71

**Published:** 2013-02-05

**Authors:** Tyler H Koep, Felicity T Enders, Chris Pierret, Stephen C Ekker, Dale Krageschmidt, Kevin L Neff, Marc Lipsitch, Jeffrey Shaman, W Charles Huskins

**Affiliations:** 1 Clinical and Translational Sciences, Mayo Graduate School, Mayo Clinic, Rochester, MN, 55905, USA; 2 Department of Health Sciences Research, Mayo Clinic, Rochester, MN, 55905, USA; 3 Department of Biochemistry and Molecular Biology, Mayo Clinic, Rochester, MN, 55905, USA; 4 Department of Facilities and Support Services, Mayo Clinic, Rochester, MN, 55905, USA; 5 Center for Communicable Disease Dynamics, Department of Epidemiology and Department of Immunology and Infectious Diseases, Harvard School of Public Health, Boston, MA, 02115, USA; 6 Department of Environmental Health Sciences, Mailman School of Public Health, Columbia University, New York, NY, 10032, USA; 7 Division of Pediatric Infectious Diseases, Department of Pediatric and Adolescent Medicine, Mayo Clinic Children’s Center, Rochester, MN, 55905, USA

**Keywords:** Influenza, Humidity, Schools, Climate

## Abstract

**Background:**

Low absolute humidity (AH) has been associated with increased influenza virus survival and transmissibility and the onset of seasonal influenza outbreaks. Humidification of indoor environments may mitigate viral transmission and may be an important control strategy, particularly in schools where viral transmission is common and contributes to the spread of influenza in communities. However, the variability and predictors of AH in the indoor school environment and the feasibility of classroom humidification to levels that could decrease viral survival have not been studied.

**Methods:**

Automated sensors were used to measure temperature, humidity and CO_2_ levels in two Minnesota grade schools without central humidification during two successive winters. Outdoor AH measurements were derived from the North American Land Data Assimilation System. Variability in indoor AH within classrooms, between classrooms in the same school, and between schools was assessed using concordance correlation coefficients (CCC). Predictors of indoor AH were examined using time-series Auto-Regressive Conditional Heteroskedasticity models. Classroom humidifiers were used when school was not in session to assess the feasibility of increasing indoor AH to levels associated with decreased influenza virus survival, as projected from previously published animal experiments.

**Results:**

AH varied little within classrooms (CCC >0.90) but was more variable between classrooms in the same school (CCC 0.81 for School 1, 0.88 for School 2) and between schools (CCC 0.81). Indoor AH varied widely during the winter (range 2.60 to 10.34 millibars [mb]) and was strongly associated with changes in outdoor AH (p < 0.001). Changes in indoor AH on school weekdays were strongly associated with CO_2_ levels (p < 0.001). Over 4 hours, classroom humidifiers increased indoor AH by 4 mb, an increase sufficient to decrease projected 1-hour virus survival by an absolute value of 30% during winter months.

**Conclusions:**

During winter, indoor AH in non-humidified grade schools varies substantially and often to levels that are very low. Indoor results are predicted by outdoor AH over a season and CO_2_ levels (which likely reflects human activity) during individual school days. Classroom humidification may be a feasible approach to increase indoor AH to levels that may decrease influenza virus survival and transmission.

## Background

Previous influenza studies have examined the association between absolute and relative humidity and influenza activity [1-3]. Absolute humidity (AH) is the water content of air, measured in millibars (mb), independent of barometric pressure and temperature. Relative humidity (RH) is the ratio (expressed as a percent) of the measured water content of air relative to the maximum possible water content of that air, which is dependent on the barometric pressure and temperature. Given these relationships, at a specified AH, the RH of colder air will be higher than that of warmer air. Recent studies indicate that absolute humidity (AH) is associated with the transmission of influenza. Animal experiments show that viral survival and transmissibility are enhanced under conditions of low AH [3]. In temperate climates, seasonal influenza epidemics and waves of the 2009 A/H1N1 influenza pandemic have been associated with decreases in outdoor AH [4,5]. Despite these findings, the role of AH in viral transmission in indoor environments—where the bulk of influenza transmission in temperate regions likely occurs during winter—has not been examined.

Grade schools are an important setting to study the association between AH and influenza transmission because large numbers of school children are exposed to similar indoor air conditions for many hours during the day and because of the prominent role that viral transmission within schools plays in the spread of influenza in communities [6,7].

In this study, we examined the variability of indoor AH within and between classrooms during winter in two grade schools in Minnesota, a state with a temperate climate including cold, dry winters. We also examined the association between hourly and daily fluctuations of indoor AH and outdoor AH and the association between indoor AH and indoor temperature, relative humidity (RH), and CO_2_ levels (as a surrogate for moisture contributions from human respiration). We then determined the effect of in-room humidification on indoor AH in classrooms. Based on these observations, we modeled the potential effects of indoor AH on influenza virus survival in classrooms.

## Methods

### Schools

The study was conducted in two grade schools in Rochester, Minnesota. These schools are participating in the Integrated Science Education Outreach (InSciEd Out) program, a partnership between the Rochester Public Schools, Mayo Clinic, and Winona State University to enhance science education in schools [8]. The two schools were chosen due to convenience, access, and the interest of school leaders in the study. The Mayo Clinic Institutional Review Board (IRB) determined that the study was not subject to IRB review because it did not involve human subjects. Physical characteristics of the schools are described in Table 1.

**Table 1:** School descriptions and ventilation capacities

	**Size in square feet (year of construction)**	**Number of rooms**	**Sensor placement**	**HVAC systems**
School 1	Main Building: 33,095 (1950)	15 classrooms	Intra-Room Variability: 5 sensors in each of 3 rooms	Air Exchange: 15 cubic feet per minute (CFM) per person
	Addition: 16,813 (1965)	7 student service rooms	Inter-Room Variability: 14 unique rooms	Heat: Steam and hot water
			Inter-School Variability: 32 combined rooms between schools	
School 2	Main Building: 145,279 (1962)	29 classrooms	Intra-Room Variability: 5 sensors in 1 room	Air Exchange: 15 cubic feet per minute (CFM) per person
	Addition: 20,704 (1989)	25 student service rooms	Inter-Room Variability: 18 unique rooms	Heat: Hot water
			Inter-School Variability: 32 combined rooms between schools	

### Equipment

Automated data loggers were used to measure temperature, relative humidity and CO_2_ continuously at 5-minute intervals (HOBO U12-012, Onset: Bourne, MA, temperature accuracy 0.35°C from 0° to 50°C, relative humidity accuracy ±2.5% from 10% to 90% RH; Telaire 7001, CO_2_ measurement accuracy ±50 ppm). All sensors are calibrated at time of manufacture. No additional validation was done prior to use. Individual home humidifiers (MoistAir MA1201, Essick Air: Little Rock, AR, whole house humidification up to 2,500 sq. ft, humidistat control) were used to raise school humidity levels on weekend days when no students or staff were present.

### Sensor placement

Temperature and RH data loggers were placed at a height of 5–7 feet in various locations in School 1 and 2 to assess variability in AH within and between classrooms (Table 1). Thirty loggers were used in School 1 and forty were used in School 2. Standardized locations of loggers for within-classroom measurements were: close to the main doorway from the hallway into the classroom; near exterior facing windows and walls; on interior walls. The standardized location of loggers for between-classroom measurements was close to the main doorway from the hallway into the classroom. CO_2_ data loggers were placed at three locations in School 1 and four locations in School 2 in both classrooms and common areas.

### Humidity modification

Moist-Air MA1201 Essick Humidifiers were placed in 3 individual classrooms on a single weekend day in February 2012, which was chosen for convenience. All manipulations were performed with classroom doors to the hallway closed and building air exchange systems off. Multiple data loggers were present in each room to determine the radius of humidification. Humidifiers were set to target levels of 60% RH and were turned off upon reaching this target. Data loggers continued recording approximately 12 hours after humidifiers were turned off. Data loggers were also present in other classrooms within the school to measure AH changes in non-humidified rooms.

### Data management and statistics

Data loggers recorded indoor temperature, RH, and CO_2_ levels at 5-minute intervals from early January to late March 2011. Data were downloaded from the loggers once a month and exported into Excel using commercial software (HOBOware, Onset: Bourne, MA).

Outdoor AH conditions, measured as vapor pressure, were derived from hourly 2-meter above-ground specific humidity and surface pressure data available from the North American Land Data Assimilation System (NLDAS) project, a joint effort of the National Oceanic and Atmospheric Administration and the National Aeronautics and Space Administration [9]. Specifically, vapor pressure, *e*, was calculated using known thermodynamic relationships [10] as

(1) $$e=\frac{\mathrm{qp}}{\varepsilon +q\left(1-\varepsilon \right)}$$

where *q* is specific humidity, *p* is surface pressure and *ε* = 0.622.

Indoor AH, again measured as vapor pressure, was calculated from indoor temperature and RH measurements. Saturation vapor pressure, *e*_*s*_*(T)*, was first calculated using the Clausius-Clapeyron equation [10]:

(2) $$e_{s}\left(T\right)=e_{s}\left(T_{0}\right)\times \exp\left[\frac{L\left(T\right)}{R_{v}}\left(\frac{1}{T_{0}}-\frac{1}{T}\right)\right]$$

where *e*_*s*_(*T*_0_) = 6.11 hPa is a reference saturation vapor pressure at *T*_*0*_ = 273.15 K, *L(T)* is the latent heat of vaporization, and *R*_*v*_ is the gas constant for water vapor. Once *e*_*s*_(*T*) is calculated, vapor pressure, *e*, is then determined as

(3) $$e=e_{s}\left(T_{0}\right)\frac{\mathrm{RH}}{100\%}$$

Concordance correlation coefficients were used to assess AH variability within classrooms (intra-room), between classrooms (inter-room), and between schools (inter-school).

Estimated 1-hour influenza virus survival (IVS) was calculated using findings derived from laboratory survival experiments [1]:

(4) $$\mathrm{IVS}=\exp\left(4.516-0.0719e\right)-1$$

Predictors of indoor AH were assessed using time-series Auto-Regressive Conditional Heteroskedasticity (ARCH) models to account for daily periodicity [11]. Auto-regression is essential in a time series model as it allows indoor AH at time, t, to be adjusted for prior measurements. In this case, we included in all models the previous two measurements of indoor absolute humidity (t-1 and t-2), as well as the prior 24 hour measurement. Conditional heteroskedasticity utilizes non-constant variance, which accounted for periods of different variance within the data. Indoor AH was predicted by two sets of models. First, using hourly data from January-March 2011, indoor AH was predicted by outdoor AH, adjusted for differences by school location, with and without adjustments for outdoor temperature, and indoor temperature (Models A-C, Table 2). Second, using 5-minute data from January 31- February 4, 2011, indoor AH was predicted by CO_2_ levels, adjusted for differences by room, with and without adjustment for indoor temperature, and for whether or not school was in session, as a proxy for the presence of students and staff in the environment (Models D-E, Table 3). Robust standard errors were used throughout. We report raw associations between physical measurements of indoor AH and associated variables; also, to assess the degree of sensitivity of indoor AH to these variables, we predicted the change in AH for a one standard deviation (SD) change in each.

**Table 2:** Daily changes in indoor AH associated with changes in outdoor AH

	**Outdoor AH**	**Outdoor Temperature**	**Indoor Temperature**
Model A^*^	0.51 [0.48, 0.54]		
Model B^*^	0.50 [0.48, 0.52]	-0.02 [-0.04, -0.002]	
Model C^*^	0.48 [0.39, 0.57]	-0.05 [-0.07, -0.02]	0.36 [0.27, 0.44]
1 SD	1.86 mb	4.89°C	0.91°C

AH, absolute humidity; ARCH, auto-regressive conditional heteroskedasticity.

*The regression coefficient shows the increase in average indoor AH (mb) associated with a 1 mb increase in outdoor AH.

ARCH model association between indoor absolute humidity (mb) and outdoor absolute humidity with and without adjustments for outdoor and indoor temperature from January to early March at 3-hour time intervals, 2011; Models A, B, C adjusted for differences by school and outdoor AH, Model B, C adjusted for outdoor temperature, Model C adjusted for indoor temperature; All coefficients statistically significant with p < .001.

**Table 3:** **Hourly changes in indoor AH associated with changes in CO**_**2**_

	**100 x CO**_2_*	**Indoor Temperature**	**School vs non-school Hours**
Model D*	0. 15 [ 0.14, 0.16]		
Model E*	0. 15 [0.15, 0.16]	-0.08 [-0.01, -0.07]	0.09 [0.07, 0.11]
1 SD	1.59 mb	1.85°C	N/A

AH, absolute humidity; ARCH, auto-regressive conditional heteroskedasticity.

*The regression coefficient shows the increase in average indoor AH (mb) associated with a 100 unit increase in CO_2_ (ppm).

ARCH model association between indoor absolute humidity (mb) and CO_2_ (ppm) with and without adjustments for indoor temperature and school vs. non-school hours at 5-minut time intervals during a representative school week in early February 2011; Models D, E adjusted for differences by room and CO_2_, Model E adjusted for indoor temperature and school vs non-school hours; All coefficients statistically significant with p < .001.

## Results

### Intra- and inter-room and inter-school variability

We explored sensor agreement for indoor AH from January through March using concordance correlation coefficients (CCC) at 3-hour time intervals; details of sensor comparisons are listed in Table 1. There was little variability in indoor AH measurements at different locations within individual classrooms (intra-room concordance correlation coefficients [CCC] >0.90 in both School 1 and 2). Greater variability was observed between classrooms in both School 1 and School 2 (inter-room CCC, 0.81 [CI: 0.76, 0.87] and 0.88 [CI: 0.84, 0.92], respectively) and between School 1 and School 2 as a whole (inter-school CCC, 0.81 [CI: 0.75, 0.88]).

### Daily and hourly variability

**Figure 1:**
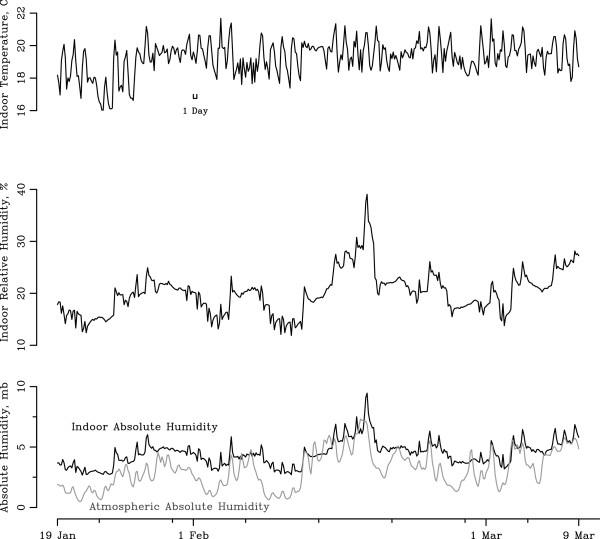
**Daily changes in winter-time absolute humidity, relative humidity, and temperature.** January 19, 2011-March 9, 2011 3 hour time series of indoor temperature, relative humidity and outdoor absolute humidity from representative sensor in School 1.

Outdoor AH, indoor AH, RH, and temperature from a representative sensor in School 1 from January to early March are displayed in Figure 1. The median outdoor AH was 4.62 mb (range: 1.83 mb to 12.50 mb). The median indoor AH was 4.86 mb (range: 2.60 mb to 10.34 mb), with low and high levels that corresponded temporally to those for outdoor AH.

Adjusting for between school differences, daily periodicity in indoor AH, and auto-regressive lags within the model, outdoor AH was associated with indoor AH over the course of the winter school period (ARCH model regression coefficient 0.51; confidence intervals [CI] 0.48, 0.54; p < 0.001; Table 2: Model A). Further adjustments for outdoor temperature and indoor temperature did not substantially modify the association between outdoor and indoor AH (Models B, C). In the adjusted model (Model C), a one standard deviation (SD) increase in outdoor AH was associated with a 0.90 mb average increase in indoor AH, while a one SD increase in outdoor and indoor temperatures were associated with a −0.24 mb and 0.33 mb average difference in indoor AH, respectively. All three coefficients were highly statistically significant in the adjusted model (p < 0.001).

**Figure 2:**
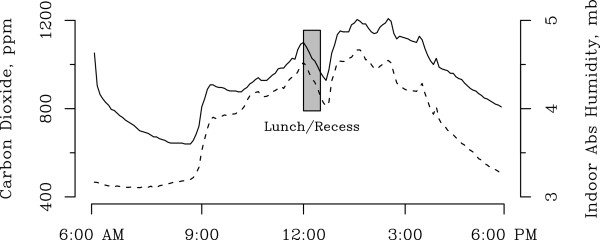
**Hourly changes in absolute humidity and school-day CO**_**2**_**.** Time series of average CO_2_ and indoor absolute humidity during the school day for the week of January 31 to February 4, 2011 in School 1. The dashed line represents mean CO_2_ levels measured every 5 minutes. The solid line is average indoor absolute humidity for the same week.

We assessed 5-minute indoor AH variations during a representative school week in early February 2011. Changes in indoor AH were closely associated with changes in CO_2_ levels during the school day (Figure 2). In contrast, measures of CO_2_ and indoor AH on weekends and holidays when staff and students were not present did not demonstrate this relationship (data not shown). We accounted for this difference by adjusting for indoor temperature and school vs. non-school hours (Model E); however, the association between CO_2_ and indoor AH was nearly identical before and after adjustment (Models D and E). In the adjusted model (Model E), a one SD increase in CO_2_ corresponded to a 0.27 mb average difference in indoor AH (p < 0.001).

### Humidity modification

**Figure 3:**
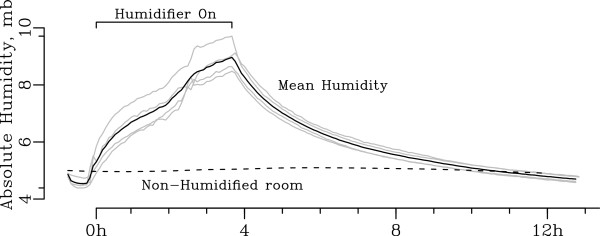
**Changes in indoor absolute humidity during classroom humidification.** Impact of humidifier on classroom indoor absolute humidity on winter weekend day in School 1. Grey lines represent 5-minute individual recordings from 4 different humidity sensors within single room. Mean absolute humidity of these sensors is shown in black. 50% of the humidification effect was gone within 2 hours post humidification.

The feasibility of increasing classroom indoor humidity with household humidifiers on weekend days is shown in Figure 3. Mean baseline indoor AH in a single room increased from 4.89 mb to a peak of 8.97 mb in a little over 4 hours. After humidification was discontinued, AH reduced 50% in 2 hours and was reduced to baseline levels in control rooms after 8 hours.

### Virus survival

**Figure 4:**
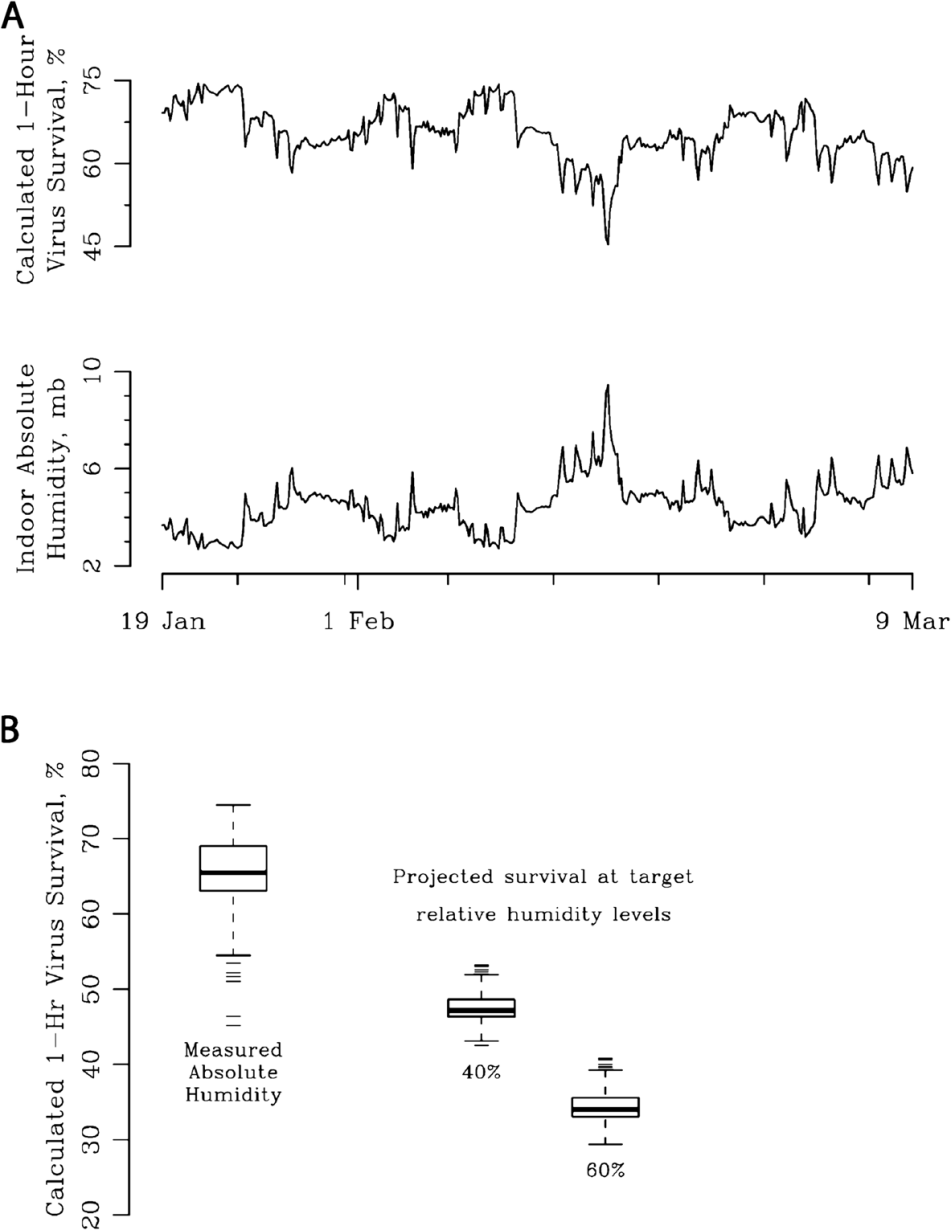
**A: Calculated daily virus survival at measured absolute humidities.** Time series of 1-hour indoor absolute humidity from representative room in School 1 and calculated 1-hour virus survival; Winter 2011. **B:** Projected virus survival at measured absolute and target relative humidities. Relationship between measured absolute humidity and projected 1-hour virus survival; Relationship between target relative humidities of 40 and 60% and projected 1-hour virus survival with the assumption of no other changes in the environment apart from humidification; Winter 2011, School 1.

Using estimates of virus survival from laboratory experiments (Equation 4) [1], maximum projected virus survival of 75% at 1 hour is observed at the lowest measured AH of 2.67 mb (Figure 4A). Conversely, peak wintertime AH values of 9.45 mb correspond to an estimated 1 hour virus survival of 45% (absolute difference: decrease in virus survival of 30%). We estimated projected virus survival changes at indoor humidification target RH levels of 40 and 60% (Figure 4B), values typically reached during non winter-time months and in our humidification experiments (Figure 3). At 60% RH, median virus survival from January through March is predicted to be 34%.

## Discussion

Indoor absolute humidity in grade schools varies substantially during winter—often to levels that are very low—and these fluctuations are associated primarily with changes in outdoor AH (Figure 1). In addition, during an individual school day, we noted that diurnal changes in indoor AH were associated closely with levels of CO_2_ (Figure 2). We speculate these diurnal changes in indoor AH are primarily due to the contribution of moisture from human respiration by students, teachers and other staff, although the use of toilets, sinks, and water fountains during weekday hours may account for a portion of these changes. Finally, we showed that classroom humidifiers can increase indoor AH substantially over several hours (Figure 3).

The implications of these findings are important with respect to the potential impact of ambient indoor AH and humidification on survival of influenza virus in the school environment. Modest, achievable changes in indoor AH are likely to have a substantial effect on 1-hour influenza virus survival, as projected from laboratory experiments (Figures 4A, 4B) [1]. For example, mean calculated 1-hour virus survival is projected to decrease from approximately 75% during periods when indoor AH is very low (~3-4 mb) to 35% when indoor AH is raised to 10 mb, a target we demonstrate can be achieved over several hours by a classroom humidifier set to a target RH of 60% on a winter day. During late spring and early fall, indoor school AH often reached 10mb and 60% RH (data not shown). Our estimation of changes in virus survival are limited to models constructed from a single set of animal experiments; however, other experiments have demonstrated similar increases in survival at very low humidity [12,13]. These data suggest that raising wintertime indoor AH to levels typically experienced indoors during fall and spring offers a strategy to reduce transmission of influenza in schools, and potentially the community, particularly when combined with vaccination and other non-pharmaceutical interventions [14-18]. As our measures reflect changes in 1 hour virus survival, humidification over a longer period (4 hours or longer) would afford a greater opportunity to reduce transmission more substantially. Indeed, this intervention may be particularly important when influenza outbreaks (or pandemics) due to novel influenza viruses occur and effective vaccines and antiviral medications are not available. However, the potential effects of wintertime humidification in older buildings need to be evaluated with respect to the potential for building rot or proliferations of mold.

Further research is needed to directly investigate the association of indoor AH with influenza virus survival in the school environment and to evaluate the effect of indoor AH on the incidence of influenza illness and school absence among school children. We also need a better understanding of school Heating Ventilation and Air Condition (HVAC) systems and their capacity for humidification, as well as additional tests of classroom humidification, to better estimate the potential benefit of humidification on decreasing influenza transmission in schools.

Our study is limited by the fact that we measured indoor AH in 2 schools using a single type of sensor in a region with very cold, dry winters. The schools were both older facilities and their HVAC systems had limited capacity for humidification. Measurement of indoor AH in newer facilities with humidification equipment may demonstrate different results. Our tests of classroom humidification were performed under controlled conditions (doors to the classroom closed and HVAC ventilation turned off), and additional study of the feasibility of classroom humidification under routine operating conditions is needed. Finally, our projections of virus survival are limited in that they are calculated using data from previously reported laboratory experiments of influenza virus survival, and we did not include direct measures of infectious virus or virus RNA [19-21]. As such, these projections serve only as an estimate of the potential impact of increasing AH in schools on influenza virus survival.

## Conclusions

We report here environmental assessments exploring the association of absolute humidity on influenza epidemiology by expanding our understanding of AH in school classrooms. We note the potential for very low indoor AH, a condition that occurs frequently during wintertime months in some regions, to increase the survival and transmission of influenza in this environment. Further investigation is necessary to determine whether increasing humidity in school classrooms is a feasible and effective means of decreasing influenza transmission among school children and in the community.

## Abbreviations

AH: Absolute Humidity; ARCH: Auto-Regressive Conditional Heteroskedasticity; RH: Relative Humidity; CCC: Concordance Correlation Coefficient; HVAC: Heating Ventilation and Air Conditioning.

## Competing interests

In the past 5 years, ML has received consulting income from the Avian/Pandemic Flu Registry (Outcome Sciences) supported in part by Roche; Novartis and Pfizer; AIR Worldwide; and i3Innovus. All of these arrangements are currently inactive. Other authors declare that they have no competing interests.

## Authors’ contributions

THK, CP, SCE, ML, JS, and WCH were involved in conception of the study. THK, FTE, DK, ML, JS and WCH contributed to study design. THK and WCH collected the data. THK, FTE, DK, KN, ML, JS, and WCH analyzed and interpreted the data. All authors drafted or critically reviewed the manuscript and approved the final version.

## Pre-publication history

The pre-publication history for this paper can be accessed here:

http://www.biomedcentral.com/1471-2334/13/71/prepub

## References

[B1] Lowen AC Mubareka S Influenza virus transmission is dependent on relative humidity and temperature PLoS Pathog 2007 3 10 1470 1476 17953482 10.1371/journal.ppat.0030151 PMC2034399

[B2] Yang W Marr LC Dynamics of airborne influenza A viruses indoors and dependence on humidity PLoS One 2011 6 6 e21481 10.1371/journal.pone.0021481 21731764 PMC3123350

[B3] Shaman J Kohn M Absolute humidity modulates influenza survival, transmission, and seasonality Proc Natl Acad Sci USA 2009 106 9 3243 3248 10.1073/pnas.0806852106 19204283 PMC2651255

[B4] Shaman J Pitzer VE Absolute humidity and the seasonal onset of influenza in the continental United States PLoS Biol 2010 8 2 e1000316 10.1371/journal.pbio.1000316 20186267 PMC2826374

[B5] Shaman J Goldstein E Absolute humidity and pandemic versus epidemic influenza Am J Epidemiol 2011 173 2 127 135 10.1093/aje/kwq347 21081646 PMC3011950

[B6] Chao DL Halloran ME School opening dates predict pandemic influenza A(H1N1) outbreaks in the United States J Infect Dis 2010 202 6 877 880 10.1086/655810 20704486 PMC2939723

[B7] Cauchemez S Ferguson NM Closure of schools during an influenza pandemic Lancet Infect Dis 2009 9 8 473 481 10.1016/S1473-3099(09)70176-8 19628172 PMC7106429

[B8] Pierret C Sonju J Leicester J Significant improvement in student proficiency: rebuilding science education InSciEd Out 2011 Washington, D.C: American Association for the Advancement of Science

[B9] NOAA/NCEP/EMC North American Land Data Assimilation System 2011 http://www.emc.ncep.noaa.gov/mmb/nldas

[B10] Wallace JM Hobbs PV Atmospheric Science, An Introductory Survey 2006 2 New York: Academic

[B11] Engle RF Lilien DM Robins RP Estimating time varying risk premia in the term structure: The ARCH-M model Econometrica 1987 55 391 407 10.2307/1913242

[B12] Schaffer FL Soergel ME Survival of airborne influenza virus: effects of propagating host, relative humidity, and composition of spray fluids Arch Virol 1976 51 4 263 273 10.1007/BF01317930 987765

[B13] Shechmeister IL Studies on the experimental epidemiology of respiratory infections J Infect Dis 1950 87 2 128 132 10.1093/infdis/87.2.128 14774528

[B14] Stebbins S Cummings DA Reduction in the incidence of influenza A but not influenza B associated with use of hand sanitizer and cough hygiene in schools: a randomized controlled trial Pediatr Infect Dis J 2011 30 11 921 926 10.1097/INF.0b013e3182218656 21691245 PMC3470868

[B15] Stebbins S Downs JS Using nonpharmaceutical interventions to prevent influenza transmission in elementary school children: parent and teacher perspectives J Public Health Manag Pract 2009 15 2 112 117 19202410 10.1097/01.PHH.0000346007.66898.67

[B16] Stebbins S Downs JS The effect of grade on compliance using nonpharmaceutical interventions to reduce influenza in an urban elementary school setting J Public Health Manag Pract 2011 17 1 65 71 21135663 10.1097/PHH.0b013e3181e83f42

[B17] Vukotich CJ Jr Coulborn RM Findings, gaps, and future direction for research in nonpharmaceutical interventions for pandemic influenza Emerg Infect Dis 2010 16 4 e2 10.3201/eid1604.090719 20350370

[B18] Lee BY Brown ST Simulating school closure strategies to mitigate an influenza epidemic J Public Health Manag Pract 2010 16 3 252 261 20035236 10.1097/PHH.0b013e3181ce594e PMC2901099

[B19] Blachere FM Lindsley WG Measurement of airborne influenza virus in a hospital emergency department Clin Infect Dis 2009 48 4 438 440 10.1086/596478 19133798

[B20] Simmerman JX Suntarattiwong P Influenza virus contamination of common household surfaces during the 2009 influenza A (H1N1) pandemic in Bangkok, Thailand: implications for contact transmission Clin Infect Dis 2010 51 9 1053 1061 10.1086/656581 20879867

[B21] Karim YG Ijaz MK Effect of relative humidity on the airborne survival of rhinovirus-14 Can J Microbiol 1985 31 11 1058 1061 10.1139/m85-199 3004682

